# Ilimaquinone and Ethylsmenoquinone, Marine Sponge Metabolites, Suppress the Proliferation of Multiple Myeloma Cells by Down-Regulating the Level of β-Catenin

**DOI:** 10.3390/md12063231

**Published:** 2014-05-27

**Authors:** Seoyoung Park, Eunju Yun, In Hyun Hwang, Soojin Yoon, Dong-Eun Kim, Ji Seon Kim, MinKyun Na, Gyu-Yong Song, Sangtaek Oh

**Affiliations:** 1 Department of Bio and Fermentation Convergence Technology, Kookmin University, Seoul 136-702, Korea; E-Mails: qkr092@nate.com (S.P.); beaver131@naver.com (J.S.K.); 2 College of Pharmacy, Chungnam National University, Daejeon 305-764, Korea; E-Mail: yunej@cnu.ac.kr; 3 Department of Chemistry, University of Iowa, Iowa City, IA 52242, USA; E-Mail: inhyun-hwang@uiowa.edu; 4 Department of Bioscience and Biotechnology, Konkuk University, Seoul 143-701, Korea; E-Mails: redsu2@naver.com (S.Y.); kimde@konkuk.ac.kr (D.-E.K.)

**Keywords:** ilimaquinone, ethylsmenoquinone, Wnt/β-catenin signaling, multiple myeloma

## Abstract

Deregulation of Wnt/β-catenin signaling promotes the development of a broad range of human cancers, including multiple myeloma, and is thus a potential target for the development of therapeutics for this disease. Here, we used a cell-based reporter system to demonstrate that ilimaquinone and ethylsmenoquinone (formerly smenorthoquinone), sesquiterpene-quinones from a marine sponge, inhibited β-catenin response transcription induced with Wnt3a-conditioned medium, by down-regulating the level of intracellular β-catenin. Pharmacological inhibition of glycogen synthase kinase-3β did not abolish the ilimaquinone and ethylsmenoquinone-mediated β-catenin down-regulation. Degradation of β-catenin was consistently found in RPMI-8226 multiple myeloma cells after ilimaquinone and ethylsmenoquinone treatment. Ilimaquinone and ethylsmenoquinone repressed the expression of cyclin D1, c-myc, and axin-2, which are β-catenin/T-cell factor-dependent genes, and inhibited the proliferation of multiple myeloma cells. In addition, ilimaquinone and ethylsmenoquinone significantly induced G_0_/G_1_ cell cycle arrest and apoptosis in RPMI-8266 cells. These findings suggest that ilimaquinone and ethylsmenoquinone exert their anti-cancer activity by blocking the Wnt/β-catenin pathway and have significant potential as therapies for multiple myeloma.

## 1. Introduction

Ilimaquinone, a sesquiterpene quinone compound, was isolated initially from *Hippospongia metachromia* [1], and it has been proposed to have a number of biological effects, including cytotoxic, anti-inflammatory and anti-viral activities [2,3,4,5]. Ilimaquinone acts through multiple pathways, such as induction of microtubule-independent Golgi fragmentation [6], up-regulation of the growth arrest and DNA damage-inducible gene 153 (CHOP/GADD153) [7], activation of hypoxia-inducible factor-1 (HIF-1) [8], and promotion of tumor necrosis factor-α (TNF-α) production [9].

Multiple myeloma, a malignant disorder of plasma cells, is the second most common hematological cancer, comprising about 10%–15% of all blood cancers [10]. It is characterized by clonal expansion of plasma cells in the bone marrow and elevation of monoclonal immunoglobulin in serum and urine. Aberrant accumulation of β-catenin, a downstream regulator of the Wnt signaling pathway, has been observed in primary multiple myeloma cells and cell lines [11]. β-Catenin is translocated into the nucleus, where it complexes with T cell factor/lymphocyte enhancer factor (TCF/LEF) family transcription factors to activate the expression of Wnt/β-catenin responsive genes, such as *c-myc*, *cyclin D1* and metalloproteinase-7 (*MMP-7*), which play important roles in tumorigenesis [12,13,14]. Thus, down-regulation of β-catenin is a potential strategy for chemoprevention and treatment of multiple myeloma. In this study, we demonstrated that ilimaquinone and ethylsmenoquinone are capable of inhibiting the Wnt/β-catenin pathway in a cell-based assay system. Ilimaquinone and ethylsmenoquinone may suppress the proliferation of multiple myeloma cells by promoting the degradation of intracellular β-catenin.

## 2. Results and Discussion

### 2.1. Ilimaquinone and Ethylsmenoquinone Suppress the Wnt/β-Catenin Pathway

**Figure 1:**
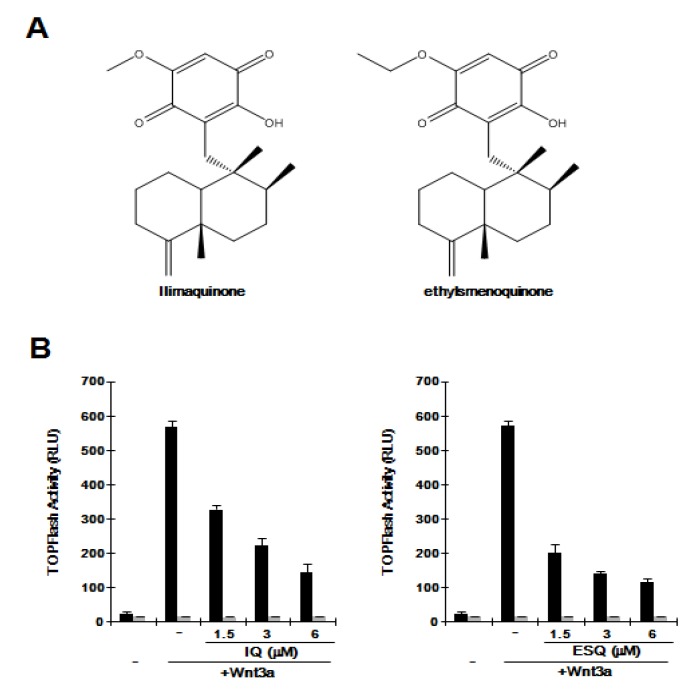
(**A**) Chemical structure of ilimaquinone (IQ) and ethylsmenoquinone (ESQ); (**B**) Concentration-dependent inhibition of β-catenin response transcription (CRT) by IQ and ESQ. HEK293-FL reporter and control cells were incubated with the indicated concentrations of IQ and ESQ in the presence of Wnt3a-CM. After 15 h, firefly luciferase activity was determined. The results represent the average of three experiments. Bars indicate standard deviations.

The Wnt/β-catenin pathway is abnormally activated in several cancers. To evaluate whether ilimaquinone and ethylsmenoquinone (Figure 1A) are capable of inhibiting this pathway, we used HEK293-FL reporter cells that were stably transfected with a synthetic β-catenin/Tcf-dependent firefly luciferase (FL) reporter and an hFz-1 expression plasmid. When HEK293-FL reporter cells were incubated with Wnt3a-conditioned medium (Wnt3a-CM), FL activity increased (Figure 1B). Addition of ilimaquinone and ethylsmenoquinone led to a dose-dependent decrease in β-catenin response transcription (CRT) activated by Wnt3a-CM; approximately 80% of CRT was suppressed with 6 μM ilimaquinone and ethylsmenoquinone (Figure 1B). In contrast, in HEK293 control cells, ilimaquinone and ethylsmenoquinone did not affect the activity of FOPFlash, a negative control reporter with mutated β-catenin/Tcf binding elements (Figure 1B), suggesting that ilimaquinone and ethylsmenoquinone are antagonists of the Wnt/β-catenin pathway.

### 2.2. Ilimaquinone and Ethylsmenoquinone Promote β-Catenin Degradation

**Figure 2:**
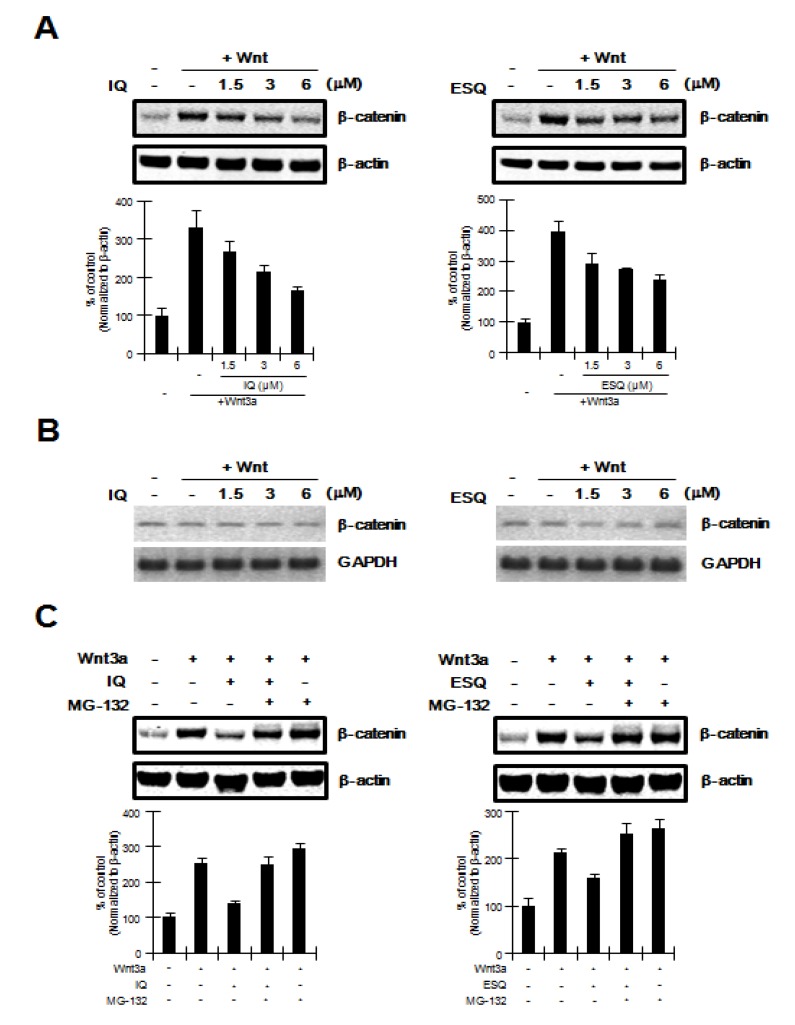
Ilimaquinone and ethylsmenoquinone promote the degradation of β-catenin via the proteasome (**A**) Cytosolic proteins were prepared from HEK293 cells treated with vehicle (DMSO) or the indicated concentrations of ilimaquinone (IQ) and ethylsmenoquinone (ESQ) in the presence of Wnt3a-CM for 15 h and then subjected to western blotting using an anti-β-catenin antibody; (**B**) Semi-quantitative RT-PCR for β-catenin and GAPDH was performed using total RNA prepared from HEK293 cells treated with vehicle (DMSO) or the indicated concentrations of IQ and ESQ in the presence of Wnt3a-CM for 15 h; (**C**) Cytosolic proteins prepared from HEK293 cells that were incubated with vehicle (DMSO) or IQ (6 μM) and ESQ (6 μM) in the presence of Wnt3a-CM were exposed to MG-132 (10 μM) for 8 h and subjected to western blotting using anti-β-catenin antibody. In (**A**), and (**C**), to confirm equal loading, the blot was reprobed with anti-actin antibody and the histogram shows the average volume density corrected for the loading control, β-actin (*n* = 3).

The Wnt/β-catenin pathway is predominantly controlled by the level of intracellular β-catenin, which is regulated by the proteasomal degradation pathway [15,16]. We therefore used western blot analysis with an anti-β-catenin antibody to examine whether ilimaquinone and ethylsmenoquinone down-regulate the intracellular β-catenin level. Consistent with results from reporter assays, treatment of HEK293 cells with ilimaquinone and ethylsmenoquinone resulted in a decrease in the amount of cytosolic β-catenin that accumulated in response to Wnt3a-CM (Figure 2A). In contrast to the protein level of β-catenin, mRNA expression of β-catenin was not affected by any of the concentrations of ilimaquinone and ethylsmenoquinone used (Figure 2B). In addition, we used MG-132, an inhibitor of the proteasome, to evaluate whether the proteasome is involved in ilimaquinone and ethylsmenoquinone-mediated β-catenin down-regulation. As shown in Figure 2C, ilimaquinone and ethylsmenoquinone consistently lowered the cytosolic β-catenin level; however, the effect of ilimaquinone and ethylsmenoquinone on the reduction of β-catenin was abolished by the addition of MG-132. Taken together, these results suggest that ilimaquinone and ethylsmenoquinone promote proteasome-mediated β-catenin degradation rather than repress β-catenin gene expression.

### 2.3. Ilimaquinone and Ethylsmenoquinone induce β-Catenin Degradation through a Mechanism Independent of GSK-3β

**Figure 3:**
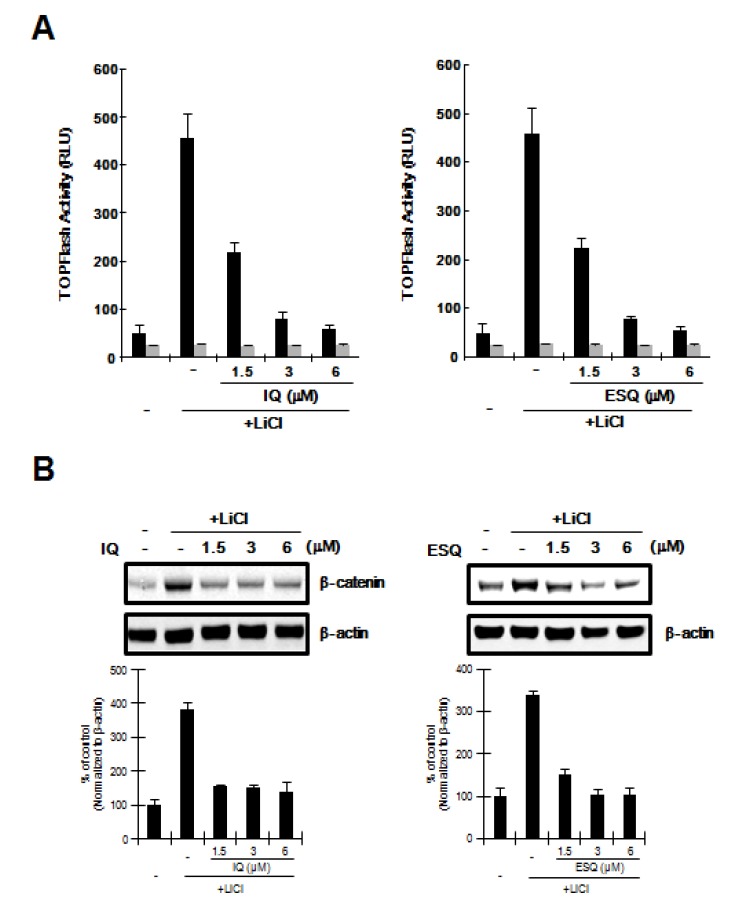
GSK-3β is not required for ilimaquinone and ethylsmenoquinone-induced β-catenin degradation. (**A**) HEK293-FL reporter cells were incubated with the indicated concentrations of ilimaquinone (IQ) and ethylsmenoquinone (ESQ) in the presence of 20 mM LiCl. After 15 h, luciferase activity was determined. The results represent the average of 3 experiments, and the bars indicate the standard deviations; (**B**) Cytosolic proteins were prepared from HEK293 cells treated with vehicle (DMSO) or IQ (1.5, 3, or 6 μM) and ESQ (1.5, 3, or 6 μM) in the presence of 20 mM LiCl for 15 h and then subjected to western blotting with an anti-β-catenin anti-body. To confirm equal loading, the blot was reprobed with anti-actin antibody. The histogram shows the average volume density corrected for the loading control, β-actin (*n* = 3).

In the Wnt/β-catenin pathway, glycogen synthase kinase-3β (GSK-3β) catalyzes the phosphorylation of β-catenin at the Ser33/37 residues, leading to its ubiquitin-dependent proteasomal degradation [17]. We used LiCl, an inhibitor of GSK-3β [18], to determine if GSK-3β activity is required for the inhibition of the Wnt/β-catenin pathway by ilimaquinone and ethylsmenoquinone. As shown in Figure 3A, incubation of HEK293-FL reporter cells with LiCl resulted in activation of CRT, but this stimulatory effect was abolished by ilimaquinone and ethylsmenoquinone. In addition, western blot analyses consistently showed that the level of intracellular β-catenin, which is up-regulated by LiCl, was lowered by treatment with ilimaquinone and ethylsmenoquinone (Figure 3B), suggesting that GSK-3β is not involved in ilimaquinone and ethylsmenoquinone-induced β-catenin degradation.

### 2.4. Ilimaquinone and Ethylsmenoquinone induce β-Catenin down-Regulation and Repress its Target Genes in RPMI-8226 Multiple Myeloma Cells

**Figure 4:**
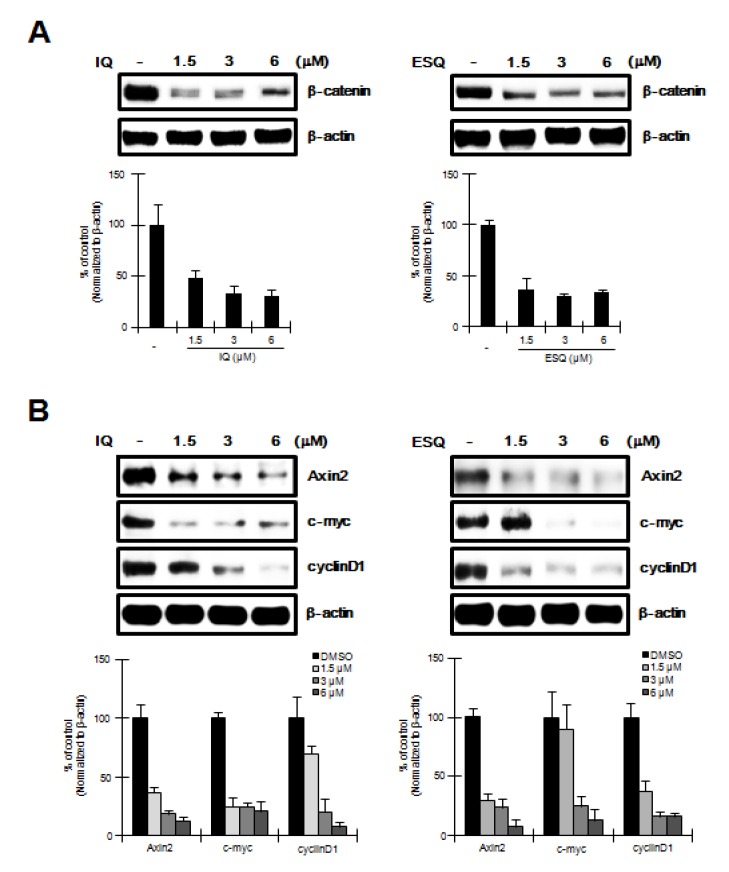
The effects of ilimaquinone and ethylsmenoquinone on the levels of β-catenin and its target genes in MM cells. (**A**) Cytosolic proteins were prepared from RPMI-8226 cells treated with vehicle (DMSO) or the indicated concentrations of IQ and ESQ for 15 h and then subjected to western blotting using an anti-β-catenin antibody.; (**B**) RPMI-8226 cells were incubated with the vehicle (DMSO) or IQ and ESQ for 15 h and then cell extracts were prepared for western blotting with anti-axin2, anti-cyclin D1 and anti-c-myc antibodies. To confirm equal loading, the blots were re-probed with anti-actin antibody and the histogram shows the average volume density corrected for the loading control, β-actin (*n* = 3).

To investigate the effect of ilimaquinone and ethylsmenoquinone on RPMI-8226 cells, in which the Wnt/β-catenin pathway is activated through constitutively active β-catenin [11], we performed western blot analyses using an anti-β-catenin antibody to determine cytoplasmic β-catenin levels in ilimaquinone and ethylsmenoquinone-treated RPMI-8226 cells. As shown in Figure 4A, ilimaquinone and ethylsmenoquinone produced a concentration-dependent decrease in cytoplasmic β-catenin levels. Because of this observation, we then examined the effect of ilimaquinone and ethylsmenoquinone on the expression of β-catenin-dependent genes in RPMI-8226 cells. After treatment of RPMI-8226 cells with various concentrations of ilimaquinone and ethylsmenoquinone, the expression levels of cyclin D1, c-myc, and axin-2, known downstream targets of β-catenin, were quantified by western blot analyses. As expected, we observed a significant decrease in cyclin D1, c-myc, and axin-2 protein levels (Figure 4B).

### 2.5. Ilimaquinone and Ethylsmenoquinone Inhibit Proliferation of RPMI-8226 Multiple Myeloma Cells

**Figure 5:**
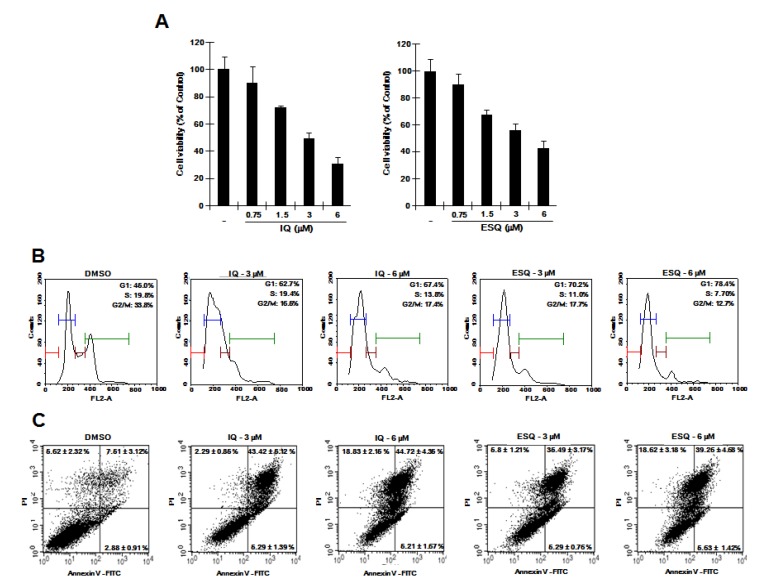
The effects of ilimaquinone and ethylsmenoquinone on RPMI-8226 cell growth. (**A**) RPMI-8226 cells were incubated for 24 h in 96-well plates with the indicated concentration of ilimaquinone (IQ) and ethylsmenoquinone (ESQ). Cell viability was examined using the CellTiter-Glo assay (Promega); (**B**) RPMI-8226 cells were incubated with the vehicle (DMSO) or IQ (3 and 6 μM) and ESQ (3 and 6 μM) for 24 h. After incubation, cells were harvested and stained with propidium iodide (PI), and analyzed using a cytometer. The x-axis indicates the PI fluorescence intensity that correlates with the DNA content; (**C**) RPMI-8226 cells were incubated with the vehicle (DMSO) or IQ (3 and 6 μM) and ESQ (3 and 6 μM) for 24 h. After incubation, cells were harvested and stained with Annexin V-FITC and PI, and analyzed using a flow cytometer. The X-axis indicates the Annexin V-FITC intensity and y-axis indicates the PI fluorescence.

Previous studies reported that specific disruption of β-catenin expression by antisense oligonucleotides or small interfering RNA suppresses the proliferation of cancer cells [19,20,21]. Given that ilimaquinone and ethylsmenoquinone down-regulate intracellular β-catenin levels, we hypothesized that they can suppress the proliferation of RPMI-8226 cells. We therefore examined the effect of ilimaquinone and ethylsmenoquinone on the growth of RPMI-8226 cells. As shown in Figure 5A, ilimaquinone and ethylsmenoquinone efficiently inhibited the proliferation of RPMI-8226 cells in a dose-dependent manner. We next investigated the possible inhibitory mechanism by analyzing cell cycle distribution using propidium iodide (PI) staining. When RPMI-8226 cells were incubated with ilimaquinone and ethylsmenoquinone, the number of cells in the S phase and G_2_/M phase was decreased, accompanied by an increase in G_0_/G_1_ cells as compared with the vehicle control (Figure 5B). In addition, the flow cytometric analyses showed that the percentage of apoptotic cells was increased by treatment with ilimaquinone and ethylsmenoquinone (Figure 5C). These results suggest that ilimaquinone and ethylsmenoquinone suppress the growth of RPMI-8226 cells by inducing G_0_/G_1_ cell cycle arrest and apoptosis.

The Wnt/β-catenin pathway plays important roles in cell proliferation, differentiation and homeostasis [22], and aberrant up-regulation of this pathway is involved in the development and progression of several human cancers. In this study, we used genetically engineered HEK293 cells, in which the Wnt/β-catenin pathway is robustly activated by Wnt3a-CM, to show that ilimaquinone and ethylsmenoquinone, marine sponge metabolites, down-regulate β-catenin through a proteasomal degradation pathway. The stability of intracellular β-catenin protein is predominantly regulated by a destruction complex composed of adenomapolyposis coli (APC), Axin, and GSK-3. The N-terminal phosphorylation of β-catenin is catalyzed by GSK-3β in a complex with APC and axin. This phosphorylated β-catenin is then recognized by the F-box β-transducin repeat-containing protein (β-TrCP), a subunit of the ubiquitin ligase complex, which results in its ubiquitin-dependent degradation [23,24]. Evidence in the present study suggests that ilimaquinone and ethylsmenoquinone induce β-catenin degradation through a mechanism independent of the destruction complex. We found that ilimaquinone and ethylsmenoquinone promoted degradation of β-catenin in the presence of Wnt3a-CM, which suppresses the function of the destruction complex. Furthermore, when GSK-3β activity was inhibited by LiCl, ilimaquinone and ethylsmenoquinone continued to destabilize intracellular β-catenin protein. Previous studies have demonstrated that β-catenin can be degraded via destruction complex-independent pathways. Liu *et al*. [25] reported that Siah-1, which is induced by p53, interacts with the carboxyl terminus of APC and then promotes the degradation of β-catenin by recruitment of the ubiquitination complex. Peroxisome proliferator-activated receptor γ and retinoid X receptor induce the degradation of β-catenin through a mechanism independent of APC and GSK-3β [26,27]. In addition, cyclin-dependent kinase 2 modulates directly β-catenin phosphorylation and degradation [28]. Previously, we demonstrated that protein kinase Cα (PKCα) catalyzes β-catenin phosphorylation at Ser33/37, thereby inducing its degradation [29]. The mechanism underlying ilimaquinone and ethylsmenoquinone-mediated β-catenin degradation needs to be investigated further.

Constitutive expression of β-catenin has been observed in MM and promotes MM cell proliferation [11]. Thus, the inhibition of β-catenin function might be an attractive strategy for treatment of MM. Small molecules and natural compounds from terrestrial origins that suppress the oncogenic function of β-catenin have been identified. PKF115-585 induces cytotoxicity in MM cells by disrupting the interaction of the transcriptionally active β-catenin/TCF complex [11]. Curcumin has been shown to stimulate caspase 3-mediated β-catenin cleavage [30] and its natural derivatives repress CRT by down-regulation of p300, a positive regulator of Wnt signaling [31]. Quercetin, a representative flavonol, suppresses Wnt/β-catenin signaling by decreasing the nuclear levels of β-catenin and Tcf4 [32], and isoflavone inactivates the Wnt/β-catenin pathway through up-regulation of GSK-3β [33]. The present study is the first to identify that ilimaquinone and ethylsmenoquinone, natural products from a marine sponge, promote β-catenin degradation and consequently repressed the expression of cyclin D1 and c-myc, which are involved in cell cycle progression and tumorigenesis, in RPMI-8226 cells.

## 3. Experimental Section

### 3.1. Isolation of Ilimaquinone

Ilimaquinone was obtained from our previous chemical investigation of three sponges *Smenospongia* *aurea*, *S. cerebriformis*, and *Verongula rigida* [34]. Briefly, dried ethanol extract (3.6 kg) of the sponges was fractionated (Fr. 1 to 13) by silica gel vacuum liquid chromatography (VLC), eluting with a stepwise gradient of hexane/acetone/methanol/water. The characteristic ^1^H-NMR signals for ilimaquinone were detected in Fr. 4, 5, 6, and 10. A gram-scale mixture of ilimaquinone and its diastereomer were isolated from the column fractions. An intensive C_18_ HPLC (250 mm × 21.2 mm, 10 μm) purification of the mixture eluting with isocratic solvent system of methanol-water (78:22) over 200 min resulted in the isolation of ilimaquinone (*t*_R_ = 136 min). ^1^H-NMR (CDCl_3_, 600 MHz): 2.08, 1.42 (each 1H, m, H_2_-1), 1.84, 1.16 (each 1H, m, H_2_-2), 2.29, 2.05 (each 1H, ddd, *J* = 13.7, 8.6, 5.4, H_2_-3), 1.49, 1.32 (each 1H, m, H_2_-6), 1.37 (2H, m, H_2_-7), 1.14 (1H, m, H-8), 0.74 (1H, d, *J* = 12.0, H-10), 4.43, 4.41 (each 1H, s, H_2_-11), 1.02 (3H, s, H_3_-12), 0.96 (3H, d, *J* = 6.4, H_3_-13), 0.82 (3H, s, H_3_-14), 2.51, 2.45 (each 1H, d, *J* = 13.7, H_2_-15), 5.83 (1H, s, H-19), 3.84 (3H, s, H_3_-22); ^13^C-NMR (CDCl_3_, 150 MHz): 23.34 (C-1), 28.11 (C-2), 33.13 (C-3), 160.69 (C-4), 40.63 (C-5), 36.82 (C-6), 28.80 (C-7), 38.25 (C-8), 43.50 (C-9), 50.30 (C-10), 102.66 (C-11), 20.73 (C-12), 18.01 (C-13), 17.52 (C-14), 32.52 (C-15), 117.49 (C-16), 153.49 (C-17), 182.51 (C-18), 102.17 (C-19), 161.90 (C-20), 182.20 (C-21), 57.01 (C-22).

### 3.2. Synthesis of Ethylsmenoquinone

To a stirred solution of ilimaquinone (400 mg) in ethanol (21 mL) was added 1M-KOH solution (10.5 mL) at room temperature. The reaction mixture was heated to 70 °C and stirred for 1 h. The reaction mixture was quenched with 1M-HCl solution and extracted with ethylacetate. The combined organic layer was washed with brine and dried over anhydrous MgSO_4_. The organic layer was concentrated *in* *vacuo*. The residue was subjected to chromatography over silica gel eluting with *n*-hexane/ethyl acetate (10:1, v/v), to give ethylsmenoquinone as a yellow semisolid (137 mg, 32.96%). ^1^H-NMR(CDCl_3_, 600 MHz): δ_H_ 7.47 (1H, s), 5.83 (1H, s), 4.46, 4.44 (each 1H, s), 4.06 (2H, q, *J* = 7.2 Hz), 2.50 (2H, dd, *J* = 12, 6.0 Hz), 2.33 (1H, dt, *J* = 12, 6.0 Hz), 2.17–1.66 (4H, m), 1.49 (3H, t, *J* = 7.2 Hz), 1.46–1.09 (7H, m), 1.04 (3H, m), 0.98 (3H, d, *J* = 6 Hz), 0.84 (3H, s); ^13^C-NMR (CDCl_3_, 150 MHz): δ_C_ 182.68, 182.26, 161.19, 160.75, 153.35, 117.45, 102.63, 102.40, 66.10, 50.35, 43.45, 40.63, 38.29, 36.82, 33.14, 32.63, 28.79, 28.12, 23.32, 20.73, 18.46, 18.05, 13.97; IT-TOF/MS: *m*/*z* = 395.2146 [M + Na]^+^. Anal. calcd. for C_23_H_32_O_4_Na.

### 3.3. Cell Culture and Chemicals

HEK293, RPMI-8226, and Wnt3a-secreting L cells were obtained from the American Type Culture Collection and maintained in DMEM supplemented with 10% fetal bovine serum (FBS), 120 μg/mL penicillin, and 200 μg/mL streptomycin. HEK293-FL reporter (TOPFlash), and control cells were established as previously described [35]. Wnt3a-conditioned medium (Wnt3a-CM) was prepared as previously described [36]. Luciferase assay was performed using the Dual Luciferase Assay Kit (Promega). LiCl, and MG-132 were purchased from Sigma-Aldrich (St. Louis, MO, USA).

### 3.4. Western Blot Analysis

Cytosolic fractions were prepared as previously described [37]. Proteins were separated by SDS-PAGE in a 4%–12% gradient gel (Invitrogen, Carlsbad, CA, USA) and transferred to nitrocellulose membranes (Bio-Rad, Hercules, CA, USA). The membranes were blocked with 5% nonfat milk and probed with anti-β-catenin (BD Transduction Laboratories, Lexington, KY, USA), anti-axin2 (Cell Signaling Technology, Denvers, MA, USA), anti-cyclin D1 (Santa Cruz Biotechnology, Santa Cruz, CA, USA), anti-myc (Santa Cruz Biotechnology), and anti-actin antibodies (Cell Signaling Technology). The membranes were then incubated with horseradish-peroxidase-conjugated anti-mouse IgG (Santa Cruz Biotechnology) or anti-rabbit IgG (Santa Cruz Biotechnology), and visualized using the ECL system (Santa Cruz Biotechnology).

### 3.5. RNA Extraction and Semi-Quantitative RT-PCR

Total RNA was isolated with Trizol reagent (Invitrogen, Carlsbad, CA, USA) in accordance with the manufacturer’s instructions. Synthesis of cDNA, reverse transcription, and PCR were performed as previously described [35]. The amplified DNA was separated on 2% agarose gels and stained with ethidium bromide.

### 3.6. Cell Viability Assay

Cells were inoculated into 96-well plates and treated with ilimaquinone and ethylsmenoquinone for 24 h. Cell viability from each treated sample was measured in triplicate by using the Celltiter-Glo assay kit (Promega, Madison, WI, USA) according to the manufacturer’s instructions.

### 3.7. Cell Cycle Analysis Viability Assay

Cells were treated with ilimaquinone and ethylsmenoquinone for 24 h. Cells were then collected, washed with cold phosphate buffered saline (PBS), and fixed in 70% ethanol at 4 °C overnight. They were then centrifuged at 2000 rpm for 5 min, resuspended in PBS, and incubated with propidium iodide (100 μg/mL) and RNase A (50 μg/mL) at room temperature for 30 min in the dark. Cells were then analyzed using a Cellometer cytometer (Nexcelom Bioscience, Lawrence, MA, USA).

### 3.8. Cell Cycle Analysis Viability Assay

After cells were treated with ilimaquinone and ethylsmenoquinone for 24 h, they were washed with cold PBS and resuspended in staining buffer containing Annexin V-FITC and propidium iodide, provided in the apoptosis detection kit (BD Transduction Laboratories), according to the manufacturer’s instructions. Flow cytometric data were obtained using a FACScalibur Flow Cytometer equipped with BD Cell Quest Pro software.

## 4. Conclusions

We investigated the anticancer effect of ilimaquinone and ethylsmenoquinone on multiple myeloma cells and revealed their mechanism of action by using a cell-based reporter system. Ilimaquinone and ethylsmenoquinone suppressed the Wnt/β-catenin pathway by promoting the degradation of β-catenin via a mechanism independent of destruction complex, thereby inhibiting the proliferation of multiple myeloma cells through inducing cell cycle arrest in G_0_/G_1_ phase and apoptosis. Ilimaquinone and ethylsmenoquinone therefore have the potential to be developed into effective cancer preventive agents and antineoplastic therapeutics for multiple myeloma.
